# Supper and Sleep

**Published:** 1887-10-08

**Authors:** 


### The Doctor's Pulpit.

### SUPPER AND SLEEP.

The people who dine at eight o'clock need not read this sermon unless they please. There is a vast number who from convenience or necessity find themselves dining at one or two, and for these the question of supper is deserving of a little intelligent consideration. The only thought many of them are inclined to give to it is expressed in the formula, "I must ask the doctor"; and if, as will sometimes happen, the doctor is not a very wise man, the answer he will give may be anything but a valuable contribution to the subject. In these days of specialism there is a tendency on the part of the public to leave everything to specialists, and to exercise their own individual judgment as little as possible. That is not merely a mistake, it is wicked idleness and intellectual cowardliness; and will inevitably meet with its just punishment in due time. Specialists are valuable persons—most valuable; but their value is generally of a limited character. In their own particular subject or range of subjects, whether that subject be snails, or crayfish, or kangaroos, or elephants, or physiologists, their words are mostly words of knowledge, and frequently words of wisdom; and he who has the opportunity should draw large cheques on their scientific banks. But there is a region in which the only infallible guide for John Smith is John Smith, and that region is the domain of his own personal experience. Of course, if John Smith be a born and utterly incapable noodle, he should ask some one to guide him even in this region. If he is not able to say whether or not he likes buttered muffins, cr whether a heavy supper of lobster salad and cheese agrees with him, then by all means let such a poor creature ask the doctor, and when he has asked him, take his advice on peril of nightmare or suicidal hypochondria. But in the case of a man of ordinary intelligence and average strength of mind the supper question is emphatically one to be decided by himself. Here, at any rate, it will be taken for granted that such a course is possible and natural; and all that we propose to do is to offer some considerations which shall guide him to a right decision.

And first—the question is one not be decided by mere abstract reasoning, or what may be called "academic physiology." Experience is a much despised, but truly great teacher. "*Experientia docet*," says one proverb: "Experience makes fools wise," says another. And experience, be it remembered, need not necessarily be merely that of the individual. There is such a thing as learning from other people's experience. Instinct, too, is a fact to be regarded. This in its pure form is hardly to be met with in man, either adult or young. But the domestic animals are at hand to be appealed to. On this question of supper, what say the horse and the cow, the cat and the dog? Do they differ in opinion and habit, or do they all agree? They are all emphatically of the same mind and practice. Each of them takes a good supper when the means are available; and it may be affirmed as a general truth that sleeplessness among them is unknown.

These animals, it may be argued, are in a state of nature. Nothing of the kind! They are domesticated and civilised according to their measure just as much as we are. When a horse is turned into a field on a summer evening the first thing he will probably do is to take a little gallop and fling up his heels; then perhaps he will lie down and "roll" for a minute or two; then up he springs and gives himself a few vigorous shakes; and then down goes his head to the grass. From that time onward he gnarls industriously at the short, sweet herbage until his sides are blown out like a barrel; and then he lies down to sleep the sleep of industry and an honest conscience. In the winter, when he is in the stable, his groom gives him a large meal about eight o'clock in the evening. Without such a meal he would stand unsatisfied in his stall; when he lay down his sleep would be restless; and when he got up in the morning he would be unrefreshed and unequal to the work of the day. There is an argument worthy of some consideration. It is not conclusive and final, but it is a help to decision.

Now take the case of individual men and women. They dine, we will say at two. At six a light meal of tea and bread-and-butter is taken. The activities of the day continue until ten or eleven. Has the stomach digested the dinner and tea completely or not? Undoubtedly both have undergone complete digestion by half-past eight, or nine at the latest. And then, as sleep has not commenced, a more or less considerable degree of hunger will assert itself. The problem is how to deal with the hunger so as to secure sound sleep and cheerful awakening. The solution is, to satisfy the appetite in such a way as to induce easy and unconscious digestion. These are the two points on which the mind must be fixed. The appetite must be satisfied, but satisfied with such a quantity and kind of food as shall be digested unconsciously. Here it is that the element of individual sense and experience is of such high value. What can you digest with comfort at ten o'clock at night? That is the question. Are you a sportsman or a working farmer, or a navvy, and do you find a pound of steak, with pickled onions, half a bread-loaf, and a foaming pint of bitter ale quite to your liking, and productive of amiability and a feeling of gentle somnolence? Then that is the supper for you. Only you do not need the occupant of the doctor's pulpit to tell you so. Your physiological morals are so sound that no preacher is of any service to you.

But how few are in this happy condition! The studious, high-strung, and worried parson would shudder at the sight of such a monstrous feast. The nervous and delicate woman whose means are limited, and whose husband and family make large demands upon her small reserves of patience and domestic skill—what will she do if she finds herself at a Friar Tuck banquet of that kind? The physiological law is made for the physiological transgressor, and the doctor's gospel of hope and help is for the weak and erring in appetite and digestion. It is very seldom that people in feeble health sleep well after a heavy supper; and it is just as likely they will have a restless night if they go to bed without any supper at all. It is impossible to tell every such person exactly what to do at all times, but as a rule this is the best kind of meal for them to take at bedtime: *a meal of rather large bulk, but very easy of digestion.* The quantity is needed to satisfy the craving of the appetite; but as the digestion, like the rest of the economy, is then fatigued and demanding sleep, the quality must be such as to allow of this function taking place with the least possible physiological effort. As an example of what is meant by a bulky meal easy of digestion, may be mentioned a large bowl of boiled bread and milk. The bread should be abundant, stale, and well soaked in the milk. This is a most satisfying supper, and highly conducive to sleep. Some people, however, cannot take milk in any form. Perhaps, in certain cases, a fair quantity of plain boiled whitefish, with one or two potatoes and stale bread, and a glass of cold or hot water or bitter ale or stout, will be found suitable. There are now in the market a large number of farinaceous preparations, both malted and unmixed. By a little taste and judgment these may be made into an almost infinite variety of bulky supper dishes. If the farinaceous food of itself be found unpalatable, veal or chicken broth, with plenty of stale bread soaked in it, as in the boiled milk, will often be relished. A common supper, and one well worthy of a trial, is a breakfast-cup of good cocoa, with plenty of bread and butter. Many people can digest the finer cheeses, such as Gruyère, Gorgonzola, Cheddar, Gloucester, etc. Those who can may be encouraged to partake of them in moderation. An excellent supper dish is tripe, with plenty of bread and a glass of light wine, such as hock or chablis. Strong wines and spirits at bedtime should generally be avoided. Some people sleep well after what they call a "night-cap," which in Scotland means a glass of hot whiskey-toddy of no mean strength; but the majority of people are far better without it. Probably no class of people sleep sounder or have better health than total abstainers: and they, as a rule, display healthy appetites at all regular meals.

Perhaps we have said enough to indicate generally whether or not suppers should be taken, and what kind of suppers they should be if they are taken. If a person has proved by experience that for him or her "supperless to bed" means sound sleep and a refreshed awakening, then by all means let such a person continue the supperless habit so long as it continues to be so satisfactory in its results. But to those who are in doubt and frequent discomfort from irregularity and uncertainty about the last meal of the day, we can recommend an intelligent and prolonged trial on the lines here laid down.

(*To be continued.*)

